# Moral Challenges When Suspecting Abuse and Neglect in School Children: A Mixed Method Study

**DOI:** 10.1007/s10560-020-00680-6

**Published:** 2020-06-16

**Authors:** M. Forsner, G. Elvhage, B. M. Ewalds-Kvist, K. Lützén

**Affiliations:** 1 Department of Nursing Sciences, Umeå University, 90187 Umeå, Sweden; 2 Department of Biosciences and Nutrition, Karolinska Institutet, Stockholm, Sweden; 3 Department of Social Work, Södertörns Högskola, Stockholm, Sweden; 4 Department of Psychology, Stockholm University, Stockholm, Sweden; 5 Department of Psychology and Speech-Language Pathology, University of Turku, Turku, Finland; 6 The Swedish Association of Health Professionals, Stockholm, Sweden

**Keywords:** Moral challenges, Report child abuse and neglect, School professionals, Social work

## Abstract

The World Health Organization (WHO), concludes that child maltreatment is a global concern calling for a multi sectoral interdisciplinary approach. School professionals, such as social workers, teachers, and health care professionals are in positions to discover and report maltreatment enabling social workers to intervene. However, a variety of reports reveal an evident gap between incidences and frequency of number of cases reported. A review of relevant research indicates that the problem of “not reporting” suggests that moral conflicts are activated in the process of decision-making. The aim was to gain a deeper understanding of school professionals’ experiences of reporting suspected neglect and abuse to the Social Welfare Board. In a mixed method approach 32 school professionals, such as teachers, social workers, nurses and psychologists participated in interviews and responded to questionnaires. Findings from the qualitative content analysis were compared to the quantitative analysis in a meta-analysis. Moral conflicts occur when faced with making decisions about how to best deal with a child’s situation. Thoughts about the child’s best interest and relationship with his/her parents as well as the informants´ own safety, were central. The comparative meta- analysis of both data sets revealed these conflicts commence with a moral sensitivity of possible negative consequences for the child. Moral sensitivity can be viewed as a “good” personal attribute, it paradoxically might lead to moral stress despite an open ethical climate. Based on the results of this study, further research on the interpersonal aspects of dealing with moral conflicts involved in reporting suspected child abuse is indicated.

The World Health Organization maintains that maltreatment of children is of a global concern. The consequences of a child being subjected to abuse and neglect, without early appropriate interventions, have adverse lifelong impact on health and social well-being (WHO, 2016). For Child Protection Services to intervene in cases of maltreatment a “multi sectoral approach” is required. There are many official sectors of professionals who are by law entrusted to observe and report any suspected childhood abuse and neglect. School is the primary sector where professionals have daily contact with pupils, enabling early detection. However, a discrepancy between the number of children exposed to neglect and abuse and the number of officially reported cases has been detected.

The question why professionals who are by law delegated to report suspected child abuse and neglect fail to do this needs to be explored. If the professionals in a school system have an accumulated knowledge to be able to identify child maltreatment and competence there must be a dimension that has yet to be explored. In this study, a focus is placed on the ethical nature of reporting suspected child abuse. From a relational ethical perspective, moral conflicts occur in situations in which one person has the authority to make decisions for another; for example, persons who are by legislation judged not to be competent or have the capacity to understand the nature and consequences of decisions that are made on their behalf. Although a country may have routines that clearly regulates the responsibility and directions in responding to suspected child maltreatment, it appears that the “human” factor has not adequately been attended to in relevant research of child maltreatment.

A personal awareness of how decisions and consequences of actions taken can be conceptualized as moral sensitivity; that one’s decision may lead to actions that may have negative outcomes for another person (Lützén, 2006). In this current study we explore school professionals’ experiences and deliberations in situation when they suspected child abuse and neglect from an ethical perspective. A mixed method approach included combining qualitative and quantitative data-collection methods, which will be further developed in following sections.

## Background

The United Nations Convention on the Rights of the Child (UN, 1989) was developed in recognition of the claim that “the child, for the full and harmonious development of his or her personality, should grow up in a family environment, in an atmosphere of happiness, love and understanding”. However, this is not always the case. According to WHO (2016) a quarter of all adults have been physically abused as children. Moreover, worldwide inequality in children’s circumstances results in child maltreatment (Gilbert et al., 2012) including all forms of physical and emotional ill-treatment (WHO, 2016). Even national inequality plays a vital role for the risk of maltreatment (Bywaters, 2015). Lundén (2011) identifies four areas of maltreatment, namely, “emotional unavailability in the parent–child relation, emotional neglect, physical neglect, and abuse” (pp. 33–34).

According to the annual report for 2016 from United Nations Children’s Fund (UNICEF) child protection systems existed in 139 countries according (UNICEF, 2017) and national mandatory legislature for reporting child maltreatment exists in many countries. This was also the case in the USA (Steen & Duran, 2014), Australia, and Canada (Mathews & Kenny, 2008) as well as most European countries according to the European Union Agency for Fundamental Rights (FRA, 2016).

Wiklund (2006) observed that neglect and physical abuse was less frequently reported in Sweden in comparison Anglo-Saxon countries. The Swedish National Board of Health (SNBH) estimated reports about maltreatment in 154/1000 children up to 17 years old during 2018. Of these 73% were 6–17 years old and approximately 75% were mandatory reports. The reports often concerned the same child (mean 1.9 reports per child) (SNBH, 2019). However, according to Cocozza, Gustafsson, and Sydsjo (2007) notifications in Sweden represent the tip of the iceberg, which is also recognized in other studies conducted in Sweden (Svensson, Andershed, & Janson, 2015; Talsma et al., 2015) as well as in other countries (Feng, Huang, & Wang, 2010; Goebbels, Nicholson, Walsh, & De Vries, 2008; Toros & Tiirik, 2016). These documented facts indicate that children’s well-being may also be seriously affected leading to lifelong consequences.

### Ethical Perspective

Research and international publications clearly address the health, social and, judicial aspects of child abuse and the need for early detection and interventions. What seems to be lacking is a discourse, grounded in empirical research, on the ethical aspects of reporting suspected child abuses. In many related studies, other concepts analogous to an ethical framework were used. Meyers and Cornille (2002) for example, found that “emotional distress” and “compassion fatigue” were commonly experienced by professionals engaged in child protection. Similarly Conrad and Kellar-Guenther (2006), found that approximately half of child-protection staff suffered from compassion fatigue.

Megan-Jane Johnstone, a scholar well-known for her work on bio-ethics, pointed out two decades ago, that the ethical aspect of child abuse has to date not received appropriate attention. According to Johnstone, “child abuse constitutes a significant moral problem and as such, demands a substantial *moral* response” (1999, p. 192). In a publication (20 years later), Johnstone maintained that despite legislation, reporting suspected child abuse to a relevant authority is inconsistent in many countries (Johnstone, 2019, pp. 352–355). She states that “reliable data is needed” in order to produce reliable interventions (p. 354), In agreement with Johnstone’s standpoint that obstructions to mandatory reporting of child abuse has not given attention to the ethical aspects, supports the need for a research design that focuses on how school professionals describe their personal involvement—feelings and actions taken-in cases of suspected maltreatment—and how they have dealt with their duty to report.

The purpose of this present study was to investigate school professionals’ experiences of dealing with suspected child abuse and neglect—and how they think, reason and act. Consequently, the following overarching research questions guided the study design:

1. What concrete incidents of reporting suspected child abuse stand out as particularly difficult for school professionals?
2. What are school professionals’ thoughts and reasoning when they decide to report or not to report suspected child abuse?
3. How can their concerns and actions taken be described as morally challenging?

## Methods

The concept of moral sensitivity, comprising, feeling, benevolence and genuineness, as described by one of the co-authors of this study, provided a theoretical framework for the study (Lützén et al., 1995). Briefly, the concept of moral sensitivity can historically be traced to the idea of “moral sense” introduced by the philosophers Hutcheson and Shaftesbury in the eighteenth century. Moral sense was viewed as an intuitive faculty that was aroused by a person-s perception of a situation as opposed to rationalist theory of ethics, that objectivity, rational and principled thinking were the only valid ways of knowing what actions to take. Hume (1990), in contrast, upheld the idea that *feelings* are required in being able to distinguish between “virtuous” and “vicious” actions. In other words, subjectivity, and a feeling for humanity was the main components of moral sense as opposed to rational and principle-based judgment. More current, the interpersonal approach to understanding morality, as a *subjective* awareness of a person’s vulnerability can be linked to a phenomenological approach to ethics. For example, Tymeiniecka (1986), introduced the Moral Sense in her work on morality from the standpoint of benevolence, intersubjectivity and context. These dimensions were also identified using the Grounded Theory research method in a psychiatric health care context in which patients had limited freedom in their own care (Lützén, Nordin & Brolin, 1994).

Timans, Wouters and Heilbron (2019) recommend the use of a mixed-method research approach in social science. In agreement with these authors, the intention of this study design was to contribute to an understanding of the moral dimension of how a selected group of participants think and act when they suspected child abuse and neglect. The mixed method design also gave a dynamic option to expand the scope and thereby improved the analytic power of the study (Levitt et al., 2018; Timans et al., 2019). The design consisted of three components: (1) Purposeful selections of participants. (2) Qualitative and quantitative data are collected simultaneously and analyzed, while the former data’s focus is on individual experience and the latter on group’s focus on means of responses relative to report child abuse and neglect. (3) Comparative analysis of both research approaches intended to validate the result of the study.

Narrative interviews can capture emotional elements of significance, whereas, quantitative data, such as the scales that were used in this study could be compared with the qualitative analysis and lead to a meta-analysis.

Thus, the mixed method design required researchers who could contribute to the analysis of data with their knowledge namely ethics, nursing, social work and psychology.

## Study Context

The Swedish Social Services Act (SFS, 2001:453) legislates that municipalities are responsible for ensuring a good and secure childhood and obliged to intervene in cases of child abuse and neglect. The municipalities through social workers, have the responsibility to intervene in cases of child abuse and neglect. Mandatory reporting to Social Welfare Board about children at risk concerns everyone working with children, including preschool, primary and secondary school. According to the Swedish Education Act (SFS, 2010:800) school attendance is mandatory for children from age 6 to 16 years. Child corporal punishment is prohibited by Swedish Parental Code (SFS, 1949:381). In January 2020 the United Nations Convention on the Rights of the Child was incorporated as Swedish Law (2018, p. 1197).

The study was performed within the primary school system in Sweden. All schools in Sweden follow government legislation as to infrastructure, level of competence of professional staff and curriculum. The Swedish Education Act (SFS, 2010:800) advocates a holistic view on educational goals as well as promotes social development and health for all pupils. Consequently, all schools are obliged to provide access to inter-professional school-health teams consisting of the school principal, nurse and doctor as well as social workers employed as counsellors. Also psychologists, and teachers with pedagogical competence in guiding children with learning difficulties are a part of the school-health team.

## Participants

The purposeful sampling was aimed at including participants with various occupations as well as a broad variation of schools within a geographic area. Six primary schools in the middle of Sweden, in rural area (n = 1), small town (n = 2) and big city (n = 3) were included, representing both public and private regime. The principal from each of the six included schools approved and distributed information about this study to all personnel. Thirty-two persons: 27 women and five men volunteered, one of them did not answer the questionnaire (Table 1). The school health team (n = 18) was represented by principals, nurses, counsellors and psychologists. The teaching staff (n = 16) was represented by teachers and auxiliary staff.

**Table 1:** Participants and their characteristics as well as number of questions in instruments used

Variable	n	M	Md	SD	Min	Max	t(30); p <
Participants	32						
Woman	27						
Man	5						
Age (years)	32	51.97	52.00	8.59	26	65	0.159 n.s
Woman	27	52.07	52.00	8.82	25	65	
Man	5	51.40	52.00	8.04	42	63	
Years in current employment	32	8.33	7.00	6.16	1	21	

Questionnaires (Questions)	N	M	Md	SD	Min	Max	Alpha^a^
Workrelaterad Moral stress	9	29.50	29.00	10.14	14	52	.852
Ethical climate	26	97.16	97.00	12.51	71	122	.862
Moral sensitivity	9	17.53	18.00	4.46	8	24	.720

^a^Cronbach’s alpha

## Data Collection

### Interviews

Face-to-face interviews were performed by combining two interview techniques, the narrative interview (Riessman, 2008) and the “think-aloud” method (Drennan, 2003). The purpose of the narrative interviews was to gain knowledge of the participants’ experience of concrete situations when they suspected abuse and neglect of a pupil. The “think aloud” interview technique was chosen to generate new perspectives, while responding to the three questionnaires.

The interviews were conducted by the first and second author. An interview guide was used to promote stability in data collected (Graneheim & Lundman, 2004). First, some background questions were asked about their professional role and experiences, followed by an open request: “Please tell about a situation in which you considered to report child abuse or neglect to Social Services”. The understanding of the decision- making process was deepened using follow-up questions such as “What happened then?” “How did you react on that?” to clarify details in the narratives. During the course of the data collection, follow up questions were supported by insights from earlier interviews. When the narrative part was exhausted the questionnaires described below were introduced with the request: “Please think aloud when you respond to the questions in the questionnaires”. The interviews lasted approximately 1 h and were recorded and thereafter transcribed verbatim.

### Questionnaires

In order to place a focus on the moral dimension of reporting child abuse and neglect, questionnaires about moral sensitivity, moral stress and ethical climate, were chosen. All three instruments have been extensively used in international studies separately or together:

*The Moral Sensitivity Questionnaire (MSQ)* Moral sensitivity in this study is conceptualized as a genuine concern for the welfare of others who are in vulnerable situations along with an awareness of the consequences of one’s actions (Lützén, 1993). Moral sensitivity is stimulated by observations and thoughts that are of moral relevance. Consequently, moral sensitivity produces feelings that have an evaluative function as to the consequence of one’s actions. The original Moral Sensitivity Questionnaire consisted of 30 items was developed by Lützén (1993) and later modified by reducing the number of items to nine (Lutzen, Dahlqvist, Eriksson, & Norberg, 2006).

*Work Related Moral Stress Questionnaire (WRMS),* was developed by Lützén et al., and is based on the supposition that a conflict between two or more alternative actions to take, causes moral stress. For instance, in situations where one person has a commitment to do what is best for the well-being of another, can lead to moral stress. An example is when a person perceives that the morally “right” thing to do is circumscribed by practical realities (Lutzen, Blom, Ewalds-Kvist, & Winch, 2010). The questionnaire contained nine statements regarding stress the participants may have experienced when concerned about the welfare of a pupil.

*The Ethical Climate Survey* (ECS) was originally developed by Olson (1998) to measure how nurses perceive the ethical climate of their workplace and translated into Swedish by Lutzen et al. (2010). The questionnaire consists of 26 items.

For our study, the questionnaires were all adapted to the school environment, i.e. mostly by changing the word “patient” to “school pupil”, leading to MSQ-S, WRMS-S, ECS-S. When responding to the questionnaires the participants were asked to think out loud, as shown in one response to a question in the Moral Stress Questionnaire: My ability to perceive pupils’ needs …”Yes, that can be the case, so I’ll put a 5 there”.

In addition, the participants completed requests for information about their occupation, age and education.

The think-aloud interviews provided information about how the questions were interpreted. The transcriptions were analyzed using the Respond Problem Matrix considering five types of problems that respondents often experience when answering questions in a survey: lexical, temporal, logical, omission/exclusion, and/or computational problem (Conrad & Blair, 1996). None of these problems were identified. On the contrary the participants expressed the questions as relevant and easy to understand.

## Analysis

### Qualitative Data Analysis

The transcribed narrative parts of the interviews were subjected to qualitative content analysis inspired by Graneheim and Lundman (2004), to show the logic in how content is abstracted, interpreted, and connected to the aim. At first, the transcriptions were read as a whole and then divided into meaning units i.e. parts in the text relating to the same central meaning. These meaning units were condensed i.e. shortened while still maintaining the core meaning. These units were coded and analyzed according to similarities and differences resulting into categories. The analysis was performed by first author in collaboration with second author. The first author was experienced in this specific method of qualitative content analysis (Forsner, Nilsson, Finnstrom, & Morelius, 2016). To strengthen trustworthiness, the analysis was discussed between authors. Furthermore, to provide transparency in the analytical process and credibility of the results, quotations from the transcribed text are provided. The transcribed interview citations were translated into English, which means that we did not correct any grammatical errors.

### Quantitative Data Analysis

The results were computed with IBM, SPSS software, versions 24. The participants’ responses to the questionnaires were subjected to descriptive statistics, principal component analysis with several high communalities (above .80) and all loading markers were set to be above .40. The occurrence of several high loading markers above .80 compensate for a smaller sample size. In agreement, a thumb rule of a ratio of 10 cases to 1 independent variable was employed in our research (Maccallum, Widaman, Zhang, & Hong, 1999). To assure the strength of the components, each component was reliability-tested by Cronbach alpha (Nunnally, 1994). For linear regression analysis this ratio is also appropriate (Tabachnik & Fidell, 1989, p. 129; Table 2, f2 = .61). By the latter method we answered the questions which predictors are valid for the moral dimensions. Spearman correlations and one-sample t-test were likewise applied when considered correct. The methods were chosen to explore relationships among variables in one sample, while keeping the confidentiality principle intact for the participants.

**Table 2:** The participants’ work-related moral stress was explained by their moral sensitivity

Model 1^a^	Unstand. coeff		Stand. coeff	t	Sig	95.0% CI(B)		Collinearity		
	B	SE	Beta			Lower	Higher	Part corr	Tolerance	VIF
(Constant)	9.49	7.47		1.271	.214	− 5.78	24.77			
Ethical climate^b^	− .05	.06	− .13	− .825	.416	− .18	.08	− .12	.93	1.1
Moral sensitivity^b^	1.43	.34	.64	4.209	.000	.73	2.12	.62 (38%)	.93	1.1

^a^Variable explained: Work-related moral stress

^b^Predictors or explanatory variables: (Constant), Ethical climate, Moral sensitivity; R^2^ = .38; f^2^= .61 > 35 (large), (Cohen, 1988)

### Meta-analysis

To fulfill the mixed-method design, both data sets was subjected to comparative meta-analysis. The narrative analysis was interpreted in light of the statistic findings in a comparative analysis. The emotional elements of significance were captured in narrative data whereas, quantitative data, such as the scales could be compared with the qualitative analysis and lead to a meta-analysis.

### Ethical Considerations

The benefits of the study were considered to outweigh any possible risk for the participants. In line with the Declaration of Helsinki, participants were assured of their rights of voluntary participation and confidentiality. Participants were assured that they could talk about their experiences without revealing identity of the persons mentioned, and that all data was going to be handle with caution to protect from identification. The participants were offered counselling if reflections on situations caused bad memories. The study was approved by the Regional Ethical Board in Uppsala, Sweden (2014/439).

## Results

### Narratives

The participants described situations in which they had considered whether to report or not when they encountered pupils who they suspected were exposed to abuse and/or neglect. Most of them recalled more than one example of abuse and neglect, which resulted in a total of 63 cases. The participants went through three phases when facing child maltreatment: (a) Awareness (b) Deliberation and (c) Aftermath. Their experiences during the phases are described in seven categories (a) feeling concerned and worrying about the child, (b) to report or not; the best for the child; maintaining the relationship with the child as well as with the parents; (c) negative consequences from actions; and longstanding concerns.

#### Awareness

Awareness appeared as *feeling concerned* and *worrying about the child*. Signs of neglect, psychological or physical, or of physical or sexual abuse raised concerns. Neglect was detected when the participants observed parental shortcomings such as lack of bonding, inability to provide adequate clothing, nutrition and security as one of the participants told: “And he had to cook his own food”. Alcohol and other drugs came up as problems among the older children. Also unauthorized school absences were recurring problems and made the participants feel concerned about the child as exemplified in this quote. “We had no idea about their home situation, but it was through the younger girl, aged 11 years, that /…/ she cried and cried and just wanted to go home and the older girl, aged 15 years, also stayed at home. But she showed no other signs”. These signs made the participants aware of possible abuse and/or neglect. Awareness also was demonstrated in experiences when worrying about the child’s wellbeing. Sensing that something wasn’t all right with the child was 'exemplified in this quote: “So everything he said was worrying me”. These kinds of episodes made the participants aware of possible maltreatment.

#### Deliberating

When deliberating whether *to report or not* and what was *the best for the child* appeared to conflict with the conviction of the importance of *maintaining the relationship with the child as well as with the parents.* Difficulties in determining what was best for the child were reported: “But this is really hard, how to think what is best for the child”. Sometimes, worries about the child were vague and reporting seemed too intrusive: “We were not sure if it was a child who was a mythomaniac or if it was true”. Some said that a negative experience from a previous situation had given them doubts that reporting would be beneficial to the child. Also, the participants felt that helping the child themselves rather than reporting was the best thing to do as one of the participants recalled: “His mother was not to count on /…/ she wants him all well but she doesn’t have the ability/…/ but there we have always tried to compensate”. In this as well as other cases, the participants said that they were against reporting and didn’t trust that the child would benefit from notifying the Social Service. Also, participants expressed a feeling that reporting could further aggravate the situation for the child as reflected in the following comments: “Then unfortunately it’s like this, we report and report. No, there’s no intervention. Everything has to be voluntary and then we see the children feeling bad and maltreated but no change in spite of the report from us. I can give several examples. And you lose, you lose motivation maybe; Yes, you know, you think twice before you report”. However, others had no hesitations, but to act and report promptly as told by one of the participants “But if someone is maltreated and is abused for different reasons then you have to intervene no matter what”. The latter was often the case when abuse was evident.

The participant wanted *to maintain a relationship with the child and/or the child’s family* and feared that notification might disrupt the relationship. The relationships with parents were emphasized as important in terms of being able to help the child. The interviews uncovered the risk of damaging parents’ trust when reporting maltreatment. The participants gave examples when parents had expressed disappointment and/or anger when they had reported child abuse and neglect to authorities. Sometimes the relationship with the parents even became hostile as told by one teacher: “That mom, she still hates me more than anything”. By contrast, some participants described the opposite, talking about a deepened relationship with the family as well as the child.

Also deliberating as to whether to report or not report, the participants told about sharing their doubts and seeking support from colleagues, school health team and the principal. Sometimes these discussions had led to reports and other times to a decision to not report to the social services. Both situations were experienced as strenuous.

#### Aftermath

In the interviews the aftermath of *negative consequences from actions* and *longstanding concerns* appeared to strongly influence the participants’ experience. Reporting to authorities could mean the loss of the child’s trust. Others told about being confronted by children who were disappointed and angry when contacted by the Social Services. Still others told about children being forbidden to have any further contact. Not attending the lessons with the teacher responsible for notification or as told by this school nurse: “The girl was forbidden to come to me and then when her sibling started at school the sibling was also not allowed to visit me”. Threats and aggressive confrontations from the parents were frequently narrated, exemplified by this participant who received a threatening telephone call from a dad: “Do you know what kind of weapons I’ve got here?” Even examples of threats to the participants’ children were reported. Also being questioned by their colleagues was described “I got a lot of crap” and that colleagues expressed conflicting opinions without being familiar with the facts. Frustration about dysfunctional collaboration with the Social Services were frequently expressed as exemplified in this quote: “I was called to the Social welfare office /…/ and this, it was about that the dad could argue and blast me down /…/ and the principal gave me support but I got no support from the social workers”.

The fact that the participants’ experiences led to *longstanding concerns* were obvious. Some of stories originated far back, one as far as twenty years ago. “But for sure it’s still with me /…/ I think a lot about how it is for the boy /…/ If he has any contact with his dad, whether it ended, whether it, like, got better”. Examples of this came up both when their concerns for a child had led to a report to the social services and in cases without notification or when the participant did not know whether someone else had reported the case. These longstanding concerns seemed to affect the participants both emotionally and morally.

### Responses to the Questionnaires

The number of questions in each employed instrument pertaining to work-related moral stress, the ethical climate and moral sensitivity, are described in Table 1. Also, the questionnaire’s internal reliability is denoted in forms of Cronbach’s alpha in Table 1.

The work-related moral stress was explained by means of a significant linear regression model (F[2.29] = 8.906, p = .001). It showed that 38% of the total variance in the work-related moral stress variable was explained by the ethical climate and professionals’ moral sensitivity. When analyzing an individual explanatory factor's significance, it was found that the ethical climate was not a significant explanatory variable to work-related moral stress. In contrast, moral sensitivity contributed to the model uniquely and significantly by explaining 38% of the variance in work-related moral stress according to the squared part correlation (Table 2).

The participants’ responses about the school’s ethical climate were reduced to four reliability-tested clusters to find out whether different clusters of the three questionnaires complemented each other. The participants’ opinions along with descriptive data as well as correlations between the clusters within the school’s ethical climate as well as with school professionals’ experience of work-related moral stress are depicted (Table 3).

**Table 3:** The Ethical Climate Survey and participants’ opinions along with correlations within survey and with work-related moral stress in school (WRMS-S)

Item cluster A, B, C & D	Spearman Rho	Cronbach alpha	M	One -	Sample	t—Test	df	*p* 2-tailed
	*p* 2-tailed			SD	95% CI	t		
(A)								
My manager is someone I can trust	A × B (df = 28)	.913	4.33	1.09	3.50–4.20	21.708	29	.000
School staff are supported and respected	.614; .001**		3.90	1.00	3.53–4.27	21.473	29	.000
My manager supports me in making decision			4.06	1.29	3.59–4.54	17.552	30	.000
My manager is someone I respect	A × C (df = 26)		4.53	.86	4.21–4.85	28.860	29	.000
School staff consults other professionals	.414; .035*		4.03	1.02	3.66–4.40	22.097	30	.000
Open conflict management			3.35	1.02	3.11–3.86	18.347	30	.000
Open climate exists, we are happy to ask			3.87	1.04	3.44–3.98	20.332	29	.000
(B)								
Different professions trust each other	B × C (df = 28)	.834	3.77	1.02	3.93–4.54	20.534	30	.000
The school is based on shared values	.529:.003**		3.84	1.00	3.86–4.48	21.304	30	.000
Different professionals respect each other			4.13	.89	3.39–3.97	25.984	30	.000
I work with competent colleagues	B × WRMS-Sa (df = 31)		4.65	.61	4.10–4.63	42.525	30	.000
The feelings of persons involved are considered	− .355; .050*		3.48	1.03	4.04–4.79	18.858	30	.000
The school staff respects one another			3.71	.74	3.44–3.98	27.946	30	.000
(C)								
My colleagues help me with difficult issues	C × D (df = 27)	.761	4.23	.82	3.93–4.54	28.374	29	.000
I participate in decisions regarding my pupils	.431; .025*		4.17	.83	3.86–4.48	27.367	29	.000
Staff have access to information necessary for handling pupils’ issues			3.68	.79	3.39–3.97	25.882	30	.000
My colleagues listen to my concerns about school problems			4.37	.72	4.10–4.63	33.293	29	.000
My manager listens to me when I talk about pupils’ problem			4.41	.98	4.04–4.79	24.190	28	.000
My manager supports me			4.61	.67	4.37–4.86	38.494	30	.000
I am not able to work as I would like to			3.35	1.17	2.93–3.78	15.959	30	.000
School policies steer difficult decisions			3.52	.89	3.19–3.84	22.006	30	.000
(D)								
My manager helps my colleagues	D × A (df = 25)	.761	3.83	.93	3.47–4.18	22.200	28	.000
Parents’ wishes are respected	.559; .004**		3.90	.70	3.65–4.16	31.036	30	.000
Staff use information about pupils			3.77	.81	3.48–4.07	26.119	30	.000
Safe schooling is given in my school	D × B (df = 27)		3.97	.71	3.71–4.23	31.276	30	.000
Parents know what to expect from the school	.465; .015*		3.59	.78	3.29–3.88	24.760	28	.000

Correlation is significant at the .05 level (2-tailed)*

Correlation is significant at the .01 level (2-tailed)**

The participants’ work-related moral stress was reduced by PCA and yielded two components. The components’ correlations with moral sensitivity in the school environment are depicted in Table 4.

**Table 4:** The participants’ Work-related Moral Stress by principal component analysis and components’ correlations with Moral Sensitivity (MSQ-S)

Principal component analysis			Spearman Rho	Cronbach	One-	Sample	t-Test	t	df	*P*
Components	A	B	*Df* = *31* *P* 2-tailed	Apha	M	SD	95% CI			2-tailed
You are mentally tired	,865		A × B	.797	2.69	1.62	2,11–3,27	9.412	31	.000
You are physically tired	,823		.614; .000**		3.81	1.55	3.25–4.37	13.877	31	.000
You think about quitting your work at school	,665				2.63	1.86	1.95–3.30	7.974	31	.000
You suffer from sleep deprivation	.650		A × MSQ-S		2.59	1.90	1.91–3.28	7.729	31	.000
You find it difficult to relax during your leisure time	.521		.431; .016*		2.78	1.64	2.19–3.37	9.588	31	.000
You have a bad conscience	.469				3.63	1.54	3.07–4.18	13.317	31	.000
You feel inept because you are unable to carry out your work		.927	B × MSQ-S	.749	3.77	1.69	3.06–4.31	12.051	31	.000
You cannot perform your duties		.730	.445; .012*		3.71	1.49	3.07–4.18	13.317	31	.000
You cannot do enough for the pupils		.546			4.19	1.40	3.68–4.71	16.672	30	.000

Extraction Method: Principal Component Analysis (PCA). Rotation Method: Oblimin with KMO = .780; Bartlett 96, df = 36, p = .000; a. Rotation converged in 6 iterations

*Correlation is significant at the 0.05

**At the 0.01 level (2-tailed)

The participants’ moral sensitivity is depicted in Table 5. Also correlations with work-related moral stress in school are denoted (Table 5).

**Table 5:** The participants’ Moral Sensitivity (MSQ-S) and its relationship to Work-related Moral Stress (WRMS-Sa & WRMS-Sb)

Items	Spearman Rho	Cronbach	One-	Sample	t-test	t	df	*p*
	*Df* = 31; p < .05, 2-tailed	alpha	M	SD	95% CI			2-tailed
I feel *a* personal responsibility for the pupils that they get a good education	MSQ-S × WRMS-Sa .431; .016	.749	5.32	.909	5.02–5.67	33.515	31	.000
I often do more than I can handle			4.45	1.457	3.92–4.99	17.012	30	.000
It is heavy to hold such feelings			3.94	1.436	3.35–4.40	15.080	31	.000
I am aware of the balance between the potential of doing good and the risk of doing harm to the pupils	MSQ-S × WRMS-Sb .445; .012		4.94	1.063	4.59–5.35	26.464	31	.000
I often end up in situations where I feel inadequate			4.10	1.620	3.39–4.61	13.430	31	.000

### Meta-analysis

**Fig. 1:**
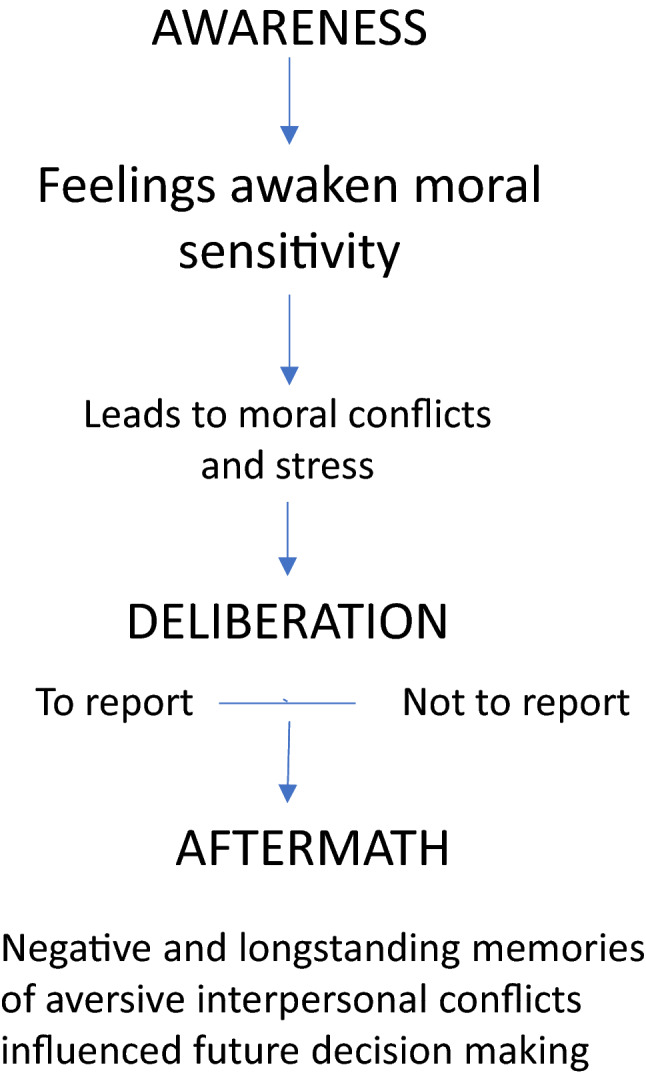
Meta-analysis of the participants experiences, thoughts, and reasoning and answers to the questionnairs; Moral sensitivity, moral stress and ethical climate

To furthermore understand the moral challenges when suspecting child abuse and neglect, the narrative data was interpreted in light of the statistic findings. Thus, expressions such as “felt concern for the child” and “to do what is best” builds on a cognitive awareness and moral motivation to do what one feels is right. The participants in our study said that their hesitation to report was in concern of the negative consequences a report may have for the child. The participants assessed their ability to perceive the needs, and to identify pupils who are in distress, as satisfactory. Their awareness of the conflict between the obligation to act and the moral awareness of the possibility of doing harm, appears to be the main conflict in this study. The interpretation of the participants’ experiences can be viewed as a decision-making process, not necessarily linear, that is initiated by feelings of concern for the welfare of the child (see Fig. 1). These feelings initiated a cognitive awareness of the consequences of decisions that will have an immediate or future impact on the child. The participants, in this study, expressed a sense of personal vulnerability; being exposed to threats and being questioned by colleagues and social service as to their observations of suspected child abuse. In some cases, the participants’ concern for the welfare of the child and lack of parental care seemed to lead to a change in their professional role, substituting their professional role for a proxy parental role.

## Discussion

The aim of this study was to gain a deeper understanding of school professionals experience when they suspect child abuse and neglect and how they think, reason and act. The findings of this study cast light on the moral complexity involved in the course of decision-making process whether to report—or not to report- child abuse and neglect to Social Services. An awareness of the child’s dependency on each decision that is made, places a pressure on the professional to make the right decision. The situations that were described by the participants clearly substantiates Johnstone’s claim that child abuse is a significant moral issue. The *word*s used by the participants in their narratives highlight the *feeling* dimension of moral sensitivity that helps a person in “knowing” what is at stake in the decision-making process (Lützén, 1993).

The participants were worried that reporting to Social Services would damage their relationship with the child, by betraying the child’s confidence and thus affecting the possibility to help the child. Similar findings came up in focus groups with Swedish school nurses (Kraft & Eriksson, 2015). The school professional’s awareness of a child’s vulnerability and of how their decision, to report or not report, can impact on the relationship to his or her parents can be interpreted as the *cognitive* dimension of moral sensitivity (Lutzen et al., 2010). Lundén (2011) found that the relationship to parents as well as support from Social Services influence the tendency to report suspected abuse. It is possible that this was also the case in the current study. Collaboration with Social Services, as well as support from colleagues, were perceived as important for the motivation to report suspected child abuse. Also, contrary negative experiences from collaboration with Social Services made school professionals disinclined to report long afterwards. The findings of Feng, Chen, Fetzer, Feng, and Lin (2012) also indicate that once teachers know about child abuse, their intention to report is most influenced by factors in their employing school. Social workers need to be aware of the importance of trusting collaboration with school professionals to facilitate their intention to report, without hesitation, every noticed sign of maltreatment and abuse.

The participants’ responses to the questionnaires show that work-related moral stress was explained by moral sensitivity and not by the ethical climate as the result of earlier research (Lutzen et al., 2010). The participant’s satisfaction with ethical climate may have contributed to the fact that thoughts of leaving their employment were not prominent. Available support and resources for ethical concerns influence the ability to endure higher levels of moral stress and still be satisfied with the job situation (Ulrich et al., 2007). Moreover, high “compassion satisfaction”, i.e., a good feeling related to the ability to help others, correlated with fewer burnout symptoms (Conrad & Kellar-Guenther, 2006). It is possible that this was also the case in the current study because the participants described engagement in teaching as well as in the pupils’ well-being. Kraft and Eriksson (2015) found that school nurses’ primary intention was to support maltreated children. This intention was also revealed in the narratives in the current study and may to some extent conflict with the intent to report.

Alvarez, Kenny, Donohue, and Carpin (2004) argue that failure to report child maltreatment leaves hundreds of thousands of children and their families without the needed interventions and, furthermore, increases the risk of further maltreatment. Furthermore, they say that the reason why professionals ignore the legal mandate to report abuse is their inability to recognize the signs, along with misunderstanding of the law and worries of negative consequences. Yet, the findings of the current study, however, suggest that the interviewed school professionals were competent to identify signs of maltreatment in their pupils and that they understood the request of the law. Apprehension of negative consequences to themselves, seemed to prohibit reporting, but most prominent barrier to reporting was fear of negative consequences for the child.

## Limitations

From a quantitative standpoint the sample size, although normally distributed, is a limitation for generalization or external validity, however fulfilling the rule of thumb of 10 cases for each independent variable. As regards generalization or external validity to other groups, we suggest that future researchers seek a larger group, when realistic. Presently, our methods were tailored for one undivided group to avoid compromising confidentiality by putting the participants at risk of identification. Namely, MacCallum et al. (1999) suggest that the minimum sample size depends upon the nature of the data itself, most remarkably on its ‘strength’. Strong data is data in which item communalities are consistently high, that components exhibit high loadings on a substantial number of items (at least three or four) and the number of factors is small. These criteria were satisfied.

From a qualitative research standpoint the sample size and purposeful sampling is satisfactory (Sandelowski, 1995; Levitt et al., 2018). However, one limitation might be that persons who volunteer to be interviewed might have had more negative experiences than those not participating, thus, biasing the findings. However, since the study aimed to specifically shed light on the participants’ situations and particularly the ethical dimensions experience, the purposeful sampling was appropriate. The mixed-method design strengthened both data sampling as well as analyses (Timans et al., 2019; Levitt et al., 2018). The study did shed light on the phenomenon from a variety of perspectives by inviting both teachers and school-team members including principals, nurses, counsellors and psychologists to participate in.

Another limitation with the study is that it is not possible to guarantee the principle of anonymity. Thus, we have adopted the principle of confidentiality by not revealing details that make it possible to identify neither the participants nor the persons mentioned in the interviews. Furthermore, face to face interviews might make the participants reluctant to share experiences casting an unkind light on their role in the situation. However we interpreted the fact that the participants disclosed situations when they didn’t decide to report as an indication that the participants were honest when narrating about their experiences.

## Implication for Social Work

In order for social workers to be able to utilize intervention methods, early detection of suspected child abuse and neglect to Child Protection Services is essential. The purpose of this study was to investigate school professionals’ experiences with perspectives from teachers, nurses and psychologists as well as social workers. We think that the findings also appear to be of importance for social workers responsible to investigate the situation and intervene in the child’s best interest. Foremost, they need insight into the situation for teachers and others who have daily contact with children and adolescents and have the professional knowledge to able detect early signs of abuse and/or neglect. Moreover, a closer collaboration with school professionals may help to increase social workers’ understanding of moral challenges when deliberating what is best for the child may result in the low frequency of reporting.

## Conclusion

Since there is a proven gap between current maltreatment and reported cases social workers are not informed and are then unable to help, leaving the abused and neglected child without early appropriate interventions. Our study revealed a chain of moral conflicts beginning with the school professionals’ moral awareness of the negative consequences of reporting suspected child abuse and neglect would bring about. Problematic interactions with Social Services, colleagues and parents seemed to serve as obstacles rather than as openings for collaboration. Although moral sensitivity can be viewed as a good characteristic, it paradoxically leads to moral stress if the prime and dominant problem is not re-solved. An unexpected finding was that some of the participants, in their struggle to decide what to do, seemed to have a closer relationship with the child, hence on the brink of leaving their professional role. Whether our interpretation of this finding is correct or not should be an imperative focus for further research. Notwithstanding that this is a small study, further research on the relational and interactive aspects of dealing with moral aspects involved in reporting suspected child abuse is required.
